# Antioxidant activity and hepatoprotective potential of *Cedrelopsis grevei* on cypermethrin induced oxidative stress and liver damage in male mice

**DOI:** 10.1186/s12906-015-0740-2

**Published:** 2015-07-25

**Authors:** Abdel-Tawab H. Mossa, Tarek M. Heikal, Meriam Belaiba, Emmanuel Guy Raoelison, Hicham Ferhout, Jalloul Bouajila

**Affiliations:** Environmental Toxicology Research Unit (ETRU), Pesticide Chemistry Department, National Research Centre (NRC), 33 El Bohouth Street (former El Tahrir St.), P.O. 12622, Dokki, Giza, Egypt; Faculté de pharmacie de Toulouse, Laboratoire des Interactions Moléculaires et Réactivité Chimique et Photochimique UMR CNRS 5623, Université de Toulouse, Université Paul-Sabatier, 118 route de Narbonne, F-31062 Toulouse, France; Laboratoire de Phytochimie et Standardisation, IMRA, BP, 3833 Antananarivo 101, Antananarivo, Madagascar; Nat’Ex Biotech. Bat 7, 55 avenue Louis Breguet, 31400 Toulouse, France

**Keywords:** C*edrelopsis grevei*, Antioxidant, Hepatoprotective, Cypermethrin, Oxidative stress, Liver damage, Mice

## Abstract

**Background:**

The liver is the most sensitive and main target organ of pesticide toxicity and damage, they play an essential role in metabolism and detoxification of pesticides. Due to these functions, hepatotoxicity continues to be among the main threats to public health, and they remain problems throughout the world. Therefore, the present study was designed to evaluate the antioxidant and hepatoprotective effects of *Cedrelopsis grevei* leaves against cypermethrin (Cyp) induced oxidative stress and liver damage in male mice.

**Methods:**

The extracts were subjected to different analyses (phenolics, tannin, flavonoids, antioxidant activity and reducing power assays). For hepatoprotective evaluation, male mice were daily exposed to Cyp and/or *C. grevei* by gavages for 28 days. Hepatoprotective effects were demonstrated by significant alterations in serum liver dysfunction biomarker enzymes, liver lipid peroxidation and antioxidant enzymes.

**Results:**

The antioxidant activity of *C. grevei* methanolic extract was the highest with an IC_50_ < 225 μg/ml by DPPH assay. The high dose of methanolic extract (300 mg/kg. b.wt.) was effective to attenuate the perturbations in the tested enzymes. Histopathological examination in the liver tissue of those mice, demonstrated that a co-administration of methanolic extract (150 & 300 mg/kg/day) showed marked improvement in its histological structure in comparison to Cyp-treated group alone and represented by nil to moderate degree in inflammatory cells.

**Conclusions:**

In view of the data of the present study, it can deduce that cypermethrin caused oxidative damage and liver dysfunction in male mice. *C. grevei* extract has protective effects on cypermethrin-induced lipid peroxidation, oxidative stress and liver damage. Results indicated that administration of *C. grevei* is useful, easy, and economical to protect humans against pesticide toxicity. The results presented here can be considered as the first information on the hepatoprotective and antioxidant properties of *C. grevei* extracts. In a future study, we will identify and investigate the components responsible for the hepatoprotective and antioxidant activities of *C. grevei.*

## Background

Pesticides have been applied in agriculture and household to protect plants, animals and human from insects and vector diseases. The negligent and random uses of pesticides can cause environmental damage, food, water contamination, and health problems (*e.g.* cancer, nerve disease, birth defects). Cypermethrin (Cyp), a class II pyrethroid pesticide, first synthesized in 1974, widely used to control many pest species in agriculture, animal breeding and the household [1]. It has been reported that Cyp residues were found in the air, on walls and furniture after three months of household treatments [2]. Cyp was accumulated in adipose tissue, brain and liver of rats [3, 4] and has hepatotoxic potential in rodents [2, 5]. It crosses the blood–brain barrier and induces neurotoxicity and motor deficits [6]. However, due to the low toxicity of pyrethroids, persistence of these insecticides in mammalian tissues may be dangerous [3].

In fact, one possible mechanism of pesticide-induced toxicity is the production of reactive oxygen species (ROS) in the cell. ROS can alter oxidant/prooxidants statues and antioxidant defense system by increasing lipid peroxidation (LPO) and depleting the antioxidants in cell (enzymatic and non-enzymatic) which leading to a condition of oxidative stress [7]. It has been reported that ROS were involved in the toxicity of organophosphate insecticides (OPIs) such as chlorpyrifos [7] and pyrethroid insecticides such as prallethrin [8, 9] and a positive correlation with the liver damage has been reported. ROS, especially superoxide anion and hydrogen peroxide, are important signaling molecules in developing and proliferating cells, but also in the induction of programmed cell death [10]. ROS are transient species due to its high chemical reactivity that leads to the LPO and a massive protein oxidation and degradation [11, 12]. The author reported that ROS cause DNA damage and strand breaks as a result of modifying purines and pyrimidines bases by superoxide anion radical (O_2_^•-^), hydrogen peroxide (H_2_O_2_), and hydroxyl radical (HO^•^).

*Cedrelopsis grevei* is an endemic species in Madagascar. It was used in traditional medicine to treat malaria, fever and fatigue [13]. Our previous study [14] showed that sixty-four components were identified in *C. grevei* essential oil by GC-MS. The major constituents were: (E)-β-farnesene (27.61 %), δ-cadinene (14.48 %), α-copaene (7.65 %) and β -elemene (6.96 %). It exhibited antioxidant activities and concentration-dependent inhibitory effects on DPPH^•^ and ABTS. Previous investigations of the trunk [15] and stem [16] bark of *C. grevei* showed that five coumarins *i.e.* norbraylin, methyl-*O*-cedrelopsin, cedrecoumarin A, scoparone and braylin were isolated. Atmaca *et al.* [17] showed that hepatoprotective effect of coumarins against oxidative stress and liver damage induced by carbon tetrachloride in male rats. In addition, phenolic compounds of *C. grevei* are playing an essential role in neutralizing free radical, quenching singlet and triplet oxygen, decomposing peroxides, stabilizing lipid peroxidation and protecting the cells against oxidative damage [18].

Currently, some synthetic antioxidant use to prevent free radical damage can induce side effects [19]. So, the dietary intake of natural products is considered very important for preventing a wide variety of diseases, including allergies, cardiovascular disease, certain forms of cancer, hepatic diseases, and inflammation, which involve free radical–mediated damage in pathologically generating processes [20]. Therefore, that is an essential research about suitable herbal drugs, that could replace the chemical ones [21]. However, the widespread use of *C. grevei* in traditional medicine stimulated us to explore its potential biological activity. To the best of our knowledge, no previous study of the antioxidant and hepatoprotective activities of *C. grevei* leaves extract have been reported. Therefore, the current study was designed to evaluate the antioxidant activity and hepatoprotective effect of *C. grevei* leaves against Cyp induced oxidative stress, lipid peroxidation and liver damage in male mice.

## Materials and methods

### Chemicals and reagents

The assay kits used for biochemical measurements of aspartate aminotransferases (AST; EC 2.6.1.1.), alanine aminotransferases (ALT; EC 2.6.1.2), alkaline phosphatase (ALP; EC 3.1.3.1), lactate dehydrogenase (LDH; EC 1.1.1.27), catalase (CAT; EC 1.11.1.6), superoxide dismutase (SOD; EC 1.15.1.1)*,* glutathione-s-transferase (GST; EC 2.5.1.18), glutathione reduced (GSH), malondialdehyde (MDA) and total protein were purchased from Biodiagnostic Company, Dokki, Giza, Egypt. Cypermethrin **(95.1 %)** was obtained from Jiangsu Yangnong Chemical Co., Ltd, China. All other chemicals used were of analytical reagent grade. All reagents were purchased from Sigma–Aldrich–Fluka (Saint-Quentin France).

### *In vitro* studies

#### Plant material and extraction

The leaves of *C. grevei* was collected from Toliara, Madagascar and identified by Benja RAKOTONIRINA (Institut malgache de recherches appliquées (IMRA), Madagascar). A voucher specimen (No. TLR 07) was deposited at the Herbarium of IMRA, Madagascar. The leaves of *C. grevei* collected in Antananarivo, Madagascar were ground to fine powder and successively extracted with solvents of increasing polarity (cyclohexane, dichloromethane, ethyl acetate, ethanol and finally methanol). Thus, 200 g of leaves powder were placed in steeping with cyclohexane (2 L) for 4 h under frequent agitation at ambient temperature and pressure. The mixture was then filtrated using Wattman Paper (GF/A, 110 mm). The solvent was evaporated by rotary evaporation under vacuum at 30 °C. Then, the same powder extracted with cyclohexane was extracted again with the next solvent, dichloromethane, under the same conditions. The same protocol was applied for ethyl acetate, ethanol and methanol. Extracts were subsequently stored in sealed and amber vials at 4 °C for the following analysis.

### Photochemical study

#### Determination of phenolic contents

The phenolic content of each extract was determined by Folin-Ciocalteu method [22]. Briefly, a mixture of diluted solution (20 μL) of each extract and 100 μL of Folin-Ciocalteu reagent (0.2 N) were prepared. The mixture was incubated for 5 min at room temperature and then 80 μL of sodium carbonate solution (75 g/L in water) was added. The absorbance was read at 765 nm, after 1 h against water blank. A standard calibration curve was plotted using gallic acid (0–300 mg/L). Results were expressed as g of gallic acid equivalents (GAE)/ Kg of dry mass.

#### Determination of flavonoids contents

The flavonoids were determined by Dowd method [22]. A mixture of diluted solution (100 μL) of each extract and 100 μL of aluminum trichloride (AlCl_3_) in methanol (2 %) were prepared. The absorbance of the mixture was measured at 415 nm, after 15 min. At the same time, a blank sample formed by methanol (100 μL) and extract (100 μL) without AlCl_3_. The results were expressed as g of quercetin equivalents (QE)/Kg of dry mass from calibration curve.

#### Determination of anthocyanin content

The absorbance of the extract was measured at 510 and 700 nm in buffers at pH 1.0 (hydrochloric acid–potassium chloride, 0.2 M) and 4.5 (acetic acid–sodium acetate, 1 M) [22] using 96-well plates. 20 μl extract solution was mixed with 180 μl of buffers and the wavelength reading was performed after 15 min of incubation. Anthocyanin contents were calculated using a molar extinction coefficient (ε) of 29600 (cyanidin-3-glucoside) and absorbance of *A* = [(*A*_510_ – *A*_700_) _pH1*.*0_ – (*A*_510_ – *A*_700_)_pH4*.*5_].

#### Determination of tannin content

Proanthocyanidins reactive to vanillin were analyzed by the vanillin method [22]. One milliliter of extract solution was placed in a test tube together with 2 mL of vanillin (1 % in 7 M H_2_SO_4_) in an ice bath and then incubated at 25 °C. After 15 min, the absorbance of the solution was read at 500 nm.

#### Antioxidant activity

The free radical scavenging activities by DPPH^●^ and ABTS^•+^ assays were evaluated according to the method cited by **Afoulous*et al.*** [14]. Total reducing capacity of *C. grevei* extracts was determined according to the method of **Oyaizu** [23]. One mL of extract at different concentrations (250–1000 μg/ml) were mixed with 2.5 ml phosphate buffer (0.2 M, pH 6.6) and 2.5 mL potassium ferricyanide [K_3_ Fe (CN)_6_] (1 %). The mixture was incubated at 50 °C for 20 min, and then a portion (2.5 ml) of TCA (10 %) was added to the mixture, which was centrifuged for 10 min at 1000 x g. The upper layer of solution (2.5 ml) was mixed with distilled water (2.5 ml) and 0.5 mL FeCl_3_ (0.1 %). Then the absorbance was measured at 700 nm. Ascorbic acid (0.5-10 μg/ml) was used as the reference compound.

### *In vivo* studies

#### Animals and treatments

Healthy adult male mice weighing 22.5 ± 1.0 g were obtained from the Animal Breeding House of the National Research Centre (NRC), Dokki, Giza, Egypt. Animals were housed in clean plastic cages in the laboratory animal room (23 ± 2 °C) on the standard pellet diet and tap water *ad-libitum*, a minimum relative humidity of 40 % and a 12 h dark/light cycle. Mice were allowed to acclimate to laboratory conditions for 7 days prior to dosing. The experimental work on mice was performed with the approval of the Animal Care & Experimental Committee, National Research Centre, Giza, Egypt, and international guidelines for the care and use of laboratory animals.

Animals were randomly divided into six groups (six mice each), control, Cyp, extract (150 and 300 mg/kg. b.wt), Cyp plus extract (150 or 300 mg/kg. b.wt) groups. Cyp and *C. grevei* extracts were administered by gavages in a fixed volume of 0.1 ml/mice daily for 28 days. Cyp was dissolved in corn oil and given via oral route at dose 13.8 mg/kg b.wt 1/10 LD_50_ [24]_._*C. grevei* extracts were dissolved in water and given via oral route at dose 150 and 300 mg/kg b.wt. Dosages were freshly prepared, adjusted weekly for body weight changes, while a control group was received corn oil.

### Sample collections

At the end of the experiment, blood samples were drawn from the retro-orbital venous plexus of the animals in glass tubes. Within 20 min of blood collection, the sera were drawn after centrifugation at 3500 rpm for 10 min at **4 °C**. The sera were kept in a deep freezer (−20 °C) for biochemical analysis. Portions of liver from all animals in each group were homogenized in 50 mM Tris–HCl buffer (pH 7.4) containing 1.15 % potassium chloride. The homogenates were centrifuged at 10,000 g for 15 min at 4 °C. The collected supernatants were used for the estimation of the activities of CAT, SOD, GST enzymes and the contents of GSH and LPO.

### Body weights and relative liver weights

During the experimental period, body weight changes of male mice were recorded weekly. At the end of treatments, the mice were sacrificed by cervical dislocation. Liver of mice was quickly removed, cleaned, weighed and used for biochemical and histological studies. Then, relative weight of liver was calculated.

### Biochemical measurements

All biochemical measurements (AST, ALT, ALP, LDH, SOD, CAT, GST, MDA, and GSH) were determined using commercial kits in accordance with manufacturers’ instructions using a spectrophotometer (Shimadzu UV–VIS Recording 2401, PC, Japan).

### Histological study

Small pieces of liver samples from each group were dissected and fixed in 10 % neutral formalin, dehydrated in ascending grades of alcohol and embedded in paraffin wax. Paraffin sections (5 μm thick) were stained for routine histological study using haematoxylin and eosin (H&E). Two slides were prepared for each mice; each slid content two sections. Ten field areas for each section were selected and examined for histopathological changes (x160) under light microscope. According to **Michael** [25], the liver fields were scored as follows: normal appearance (−), minimal cellular disruption in less than 1 % of field area (+), mild cellular disruption of 1-30 % of field area (++), moderate cellular disruption of 31-60 % of field area (+++), severe cell disruption of 61-90 % of field area (++++) and very severe cellular disruption of 91-100 % of field area (++++).

### Statistical analysis

The results were expressed as means ± S.D. All data were done with the Statistical Package for Social Sciences (SPSS 17.0 for windows). The results were analyzed using one way analysis of variance (ANOVA) followed by Duncan’s test for comparison between different treatment groups. Statistical significance was set at *P* ≤ 0.05.

## Results

The highest yield of *C. grevei* was obtained with the dichloromethane extract (4.6 %) of the plant material. While, the low yield was observed with the ethyl acetate extract (0.44 %). This variation in the yields of different extracts can be explained by the difference of polarity for compounds present in the plant (Table 1). Methanolic and ethanolic extracts of *C. grevei* were found to have high total phenolic content, 41.67 ± 0.19 and 39.12 ± 0.44 g GAE/Kg ES, respectively (Table 2). So, in the present study, the polar extracts containing the highest amount of phenolic (EtOH and MeOH) were selected for the further analysis. The ethanol extract is the richest by tannins with 22.14 ± 1.03 g EC/kg ES, followed by methanol extract with 12.69 ± 0.63 g EC/Kg ES. The ethanolic and methanol extracts have obtained intermediate values for anthocyanins (respectively 1.86 ± 0.13 and 1.35 ± 0.08 g C3GE/Kg ES) (Table 2), while flavonoids not detected.

**Table 1:** Solvents extract and yield of *Cedrelpsis grevei*

Solvents	Cyclohexane	Dichloromethane	Acetate ethyl	Ethanol	Methanol
N	1	3	2	2	1
Yield (g/100 g)	2.1	4.6	0.44	2.2	0.8

N, number of extractions; *C. grevei* leaves weights 200 g dry weight; solvent = 2 liter; extraction after 4h of steeping

**Table 2:** Chemical composition of *C. grevei* leaves extracts

Extract	Phenolics (g GAE/Kg ES)	Flavonoids (g QE/Kg ES)	Tannins (g CE/Kg ES)	Anthocyanins (g C3GE/Kg ES)
Ethanol	39.12±0.44^b^	nd	22.14±1.03^a^	1.86±0.13^b^
Methanol	41.67±0.19^a^	nd	12.69±0.63^b^	1.35±0.08^d^

Nd: not detected

**Fig. 1:**
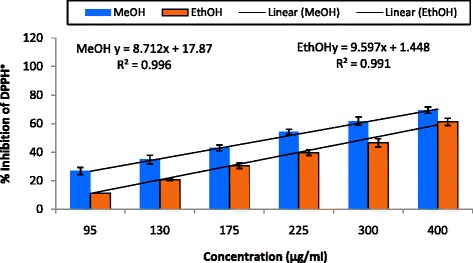
DPPH radical scavenging activity (%) of MeOH and EthOH extracts of *C. grevei*. Bars represent the mean ± SD

**Fig. 2:**
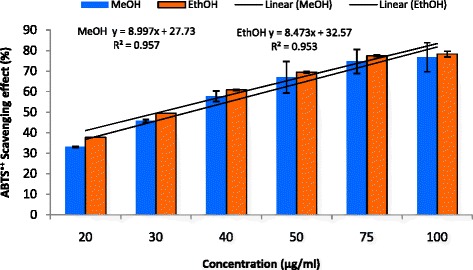
ABTS^•+^ Scavenging activity (%) of MeOH and EthOH extracts of C*. grevei*. Bars represent the mean ± SD

**Fig. 3:**
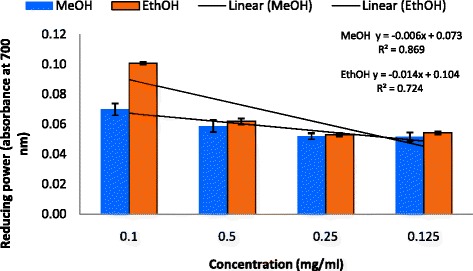
Reducing power (absorbance at 700 nm) of MeOH and EthOH extracts of *C. grevei*. Values are mean ± SD

Figure 1 illustrates a decrease in the concentration of DPPH radical due to the scavenging ability of *C. grevei* extracts. On DPPH radical, EtOH and MeOH had scavenging effects with increasing concentration in the range of 95–400 μg/mL, and the scavenging effect of EtOH was lower than MeOH. The IC_50_ value of ascorbic acid was 4.7 μg/mL. MeOH extract (400 μg/mL) exhibited the highest inhibition of about 69.45 %, but this is lower in EtOH extract whose percentage of inhibition is 61.27 %. ABTS assay is shown in Fig. 2. *C. grevei* extracts (100 μg/mL) exhibited the highest inhibition of 76.81 % and 78.35 % of MeOH and EtOH extracts. As illustrated in Fig. 3, Fe^3+^ was transformed to Fe^2+^ in the presence of MeOH and EtOH extracts of *C. grevei* to measure the reductive capability. *C. grevei* extracts had significant inhibition of reducing power with increasing concentration in the range of 0.10-0.125 mg/mL.

**Fig. 4:**
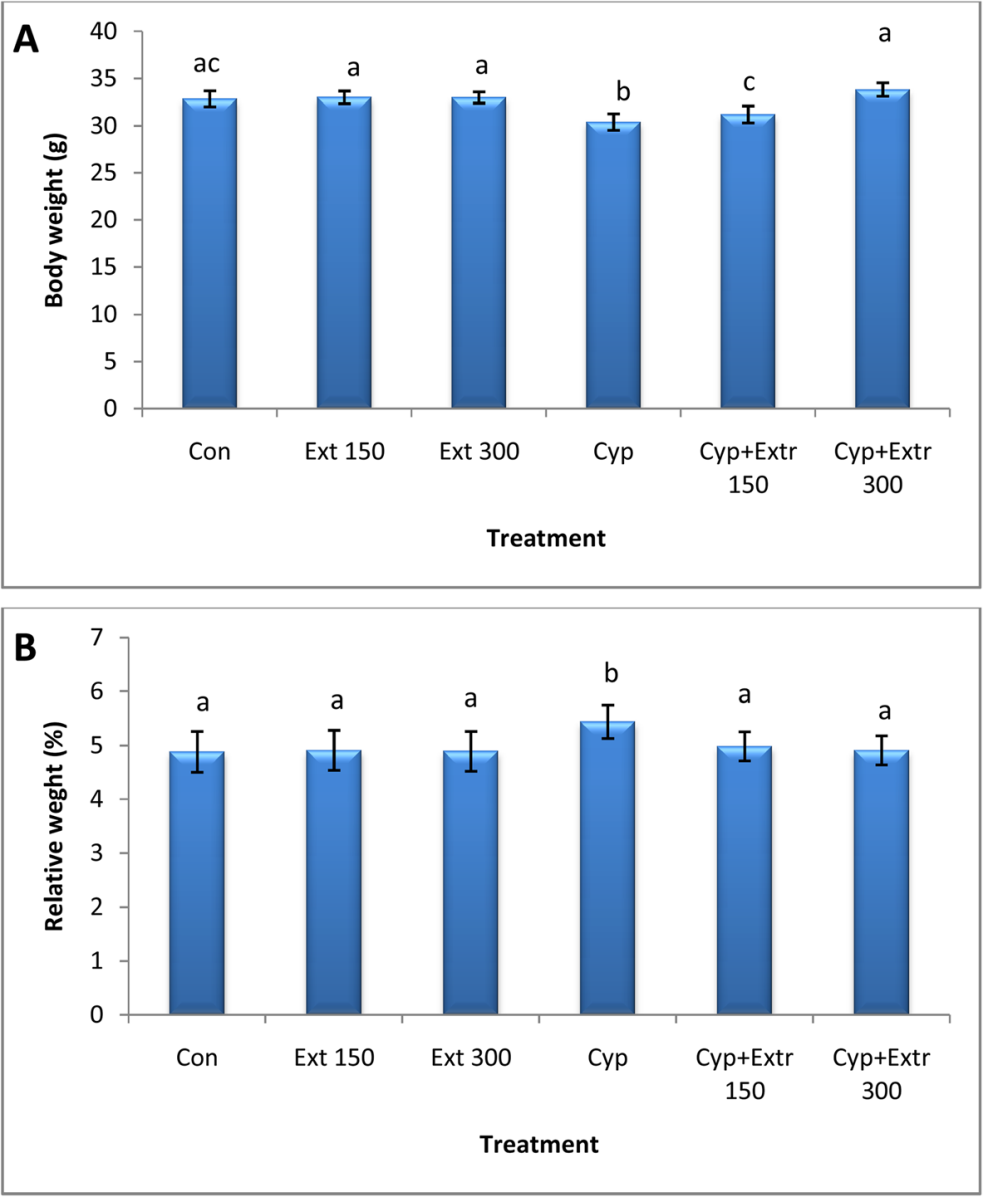
Body (**a**) and relative liver (**b**) weights of rats exposed to cypermethrin (Cyp) and the protective effect of *C. grevei* (MeOH extract). Bars represent the mean of 6 mice ± SD; values are not sharing superscripts letters (a, b, c, d, e) differ significantly at *P* ≤ 0.05. Relative liver weight (%) = (liver weight/body weight) X 100. Con: control; Cyp: cypermethrin; Ext 150: 150 mg/kg b.wt of C*. grevei* extract; Ext 300: 300 mg/kg b.wt of *C. grevei* extract (MeOH extract)

In the present study, the body weight loss was markedly observed of mice treated with Cyp compared to the control group (Fig. 4A). The difference between the two groups was statistically significant (30.38 g vs. 32.82 g, *P* ≤ 0.05). Co- administration of *C. grevei* (MeOH extract) at 300 mg/kg b.wt. to mice of Cyp group restored body weight to normal range (33.81 g vs. 32.82 g). As shown in Fig. 4B, significant decrease in relative liver weight was observed after treatment of mice with Cyp compared to control group (5.44 % g vs. 4.88 % g). Co-administration of *C. grevei* at 150 & 300 mg/kg b.wt. with Cyp modulated significantly relative live weight to the normal control value (4.98 %, 4.91 % vs. 4.88 %).

The oral administration of the two tested doses of C*. grevei* extract (150 & 300 mg/kg/day) to normal mice produced no changes in all serum biochemical parameters of liver compared to the normal control mice (Table 3). In contrast, male mice exposed to Cyp (13.80 mg/kg/day) induced a severe hepatic damage in serum enzyme activities of ALT, AST and ALP levels comparable to control (Table 3). Also in Cyp-treated mice, serum LDH activity was increased by 37.53 %, while it was decreased by −27.84 % in the liver homogenate. However, co-administration of *C. grevei* extract to Cyp-treated mice resulted in a partial recovery in the above-mentioned parameters (AST, ALT, ALP and LDH) in a dose dependent manner. The high dose of *C. grevei* extract (300 mg/kg) was more effective than low dose (150 mg/kg) to attenuate the perturbations in the tested enzymes (Table 3).

**Table 3:** Effect of *C. grevei* MeOH extract on SOD, CAT, GST, LPO and GSH in liver of mice exposed to cypermethrin

Treatments	SOD(U/g tissue))	CAT(U/g tissue)	GST(U/g tissue)	LPO(nmol/g tissue)	GSH(mmol/g tissue)
Control	664.20±8.58^c^	298.68±4.47^c^	2018.51±63.92^c^	295.42±8.12^a^	155.11±3.02^c^
Ext 150	668.74±17.49^c^	292.59±8.34^c^	2093.45±31.66^c^	298.03±9.45^a^	157.18±2.85^c^
Ext 300	676.82±20.47^c^	297.95±5.17^c^	2157.52±32.78^c^	292.38±7.25^a^	159.99±3.07^c^
Cyp	527.93±19.46^a^	183.67±6.38^a^	1642.73±52.93^a^	446.19±13.78^c^	108.48±3.93^a^
Cyp+ Ext 150	609.69±17.84^b^	272.15±6.78^b^	1710.54±53.95^ab^	409.15±20.25^c^	120.03±6.18^a^
Cyp+ Ext 300	632.40±22.02^bc^	287.87±9.92^bc^	1808.70±52.66^b^	369.50±14.58^b^	135.57±4.39^b^

Each value is a mean of 6 animals ± SD.; ^a, b, c, d^values are not shared superscripts letters (a, b, c, d) differ significantly at *P*≤ 0.05; Con: control; Cyp: cypermethrin; Ext 150 & Ext 300; 150 and 300 mg/ kg b.wt of C*. grevei* extract. SOD: superoxide dismutase; CAT: catalase; GST: glutathione-S-transferase; LPO: lipid peroxidation; GSH: glutathione reduced

In the liver homogenates of Cyp-treated mice, the activities of SOD, CAT, GST and the level of GSH decreased significantly (*P* ≤ 0.05) by 20.5 %, 38.5 %, 18.6 % and 30.1 %, respectively, while the level of MDA increased significantly (*P* ≤ 0.05) by 51.0 % when compared to the corresponding controls (Table 4). In this study, MDA level increased in mice exposed to Cyp. Co-administration of *C. grevei* extract to Cyp-treated mice resulted in a partial recovery in the above-mentioned parameters in a dose dependent manner. The high dose of C*. grevei* extract (300 mg/kg) was more effective than low dose (150 mg/kg) to attenuate the perturbations in the tested enzymes (Table 4).

**Table 4:** Effect of *C. grevei* MeOH extract on the activity of ALT, AST, ALP and LDH in serum and LDH in liver homogenate of mice exposed to cypermethrin

Treatments	ALT	AST	ALP	LDH	
	(U/L)	(U/L)	(U/L)	Serum(U/L)	Liver(U/g tissue)
Control	44.36±0.92^a^	53.33±0.32^a^	89.88±1.77^a^	176.83±6.96^a^	158.30±11.83^b^
Ext 150	45.23±0.92^a^	52.82±0.24^a^	90.97±1.29^a^	172.87±8.38^a^	160.55±10.32^b^
Ext 300	43.84±1.02^a^	53.45±0.57^a^	88.99±1.80^a^	178.32±10.05^a^	161.90±8.98^b^
Cyp	60.62±1.43^c^	69.39±2.33^c^	131.45±3.32^d^	243.21±8.23^c^	114.23±4.86^a^
Cyp+ Ext 150	55.88±1.26^b^	63.88±1.32^b^	113.84±3.05^c^	222.61±8.95^bc^	115.04±4.91^a^
Cyp+ Ext 300	53.16±1.18^b^	60.70±2.23^b^	103.10±2.25^b^	203.32±7.88^b^	133.12±2.92^a^

Each value is a mean of 6 animals ± S.E.; ^a, b, c, d^values are not shared superscripts letters (a, b, c, d) differ significantly at *P*≤ 0.05; Con: control; Cyp: cypermethrin; Ext 150 & Ext 300; 150 and 300 mg/ kg b.wt of *C. grevei* MeOH extract. ALT: alanine aminotransferases; AST: aspartate aminotransferases; ALP: alkaline phosphatase; LDH: lactate dehydrogenase

**Fig. 5:**
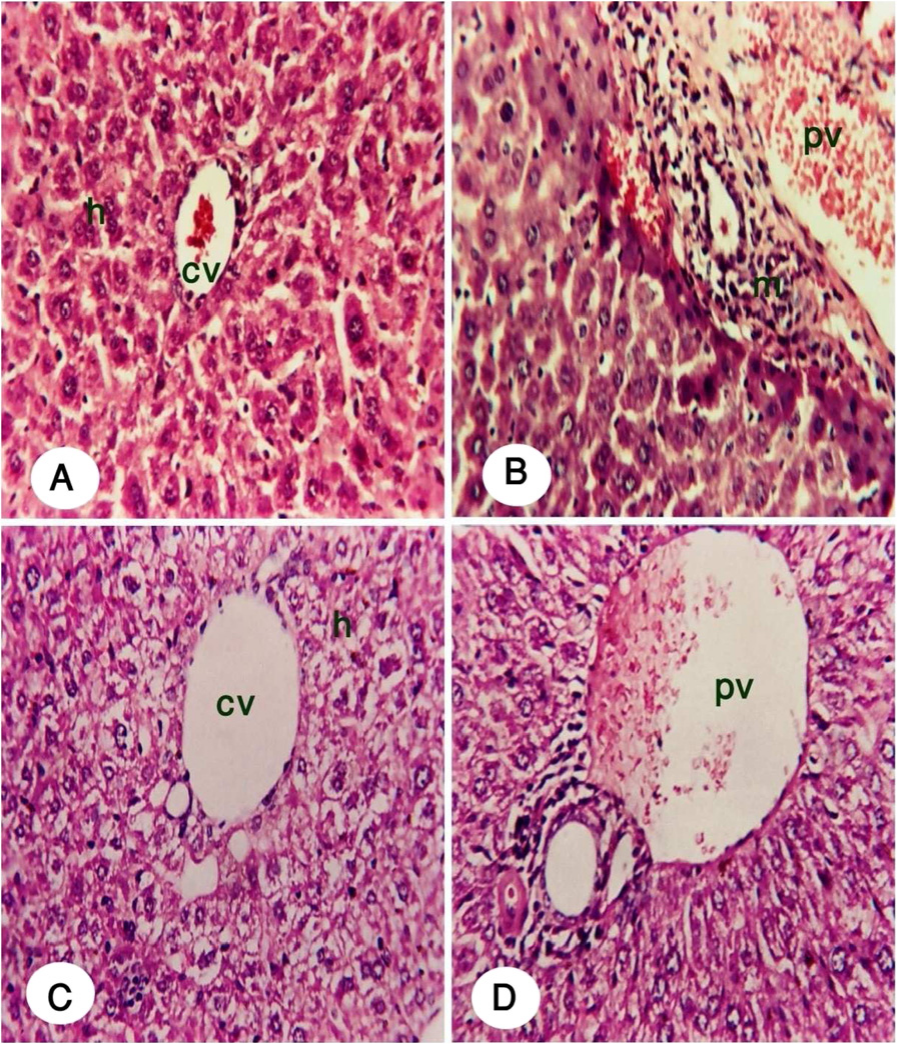
Paraffin sections of liver stained by haematoxylin and eosin (H&E) for histopathological changes. Control group and extract groups [A] showing the normal histological structure of the central vein (CV) and surrounding hepatocytes (H) (x40). Cypermethrin group showing [B] degeneration in hepatocytes and dilation of portal vein with inflammatory cells infiltration (m) in the portal area (x80). Cypermethrin plus extract at 150 mg/kg. b.wt. [C] showing dilatation of central vein (CV) ballooning degeneration (h) in surrounding hepatocytes (x80). Cypermethrin plus extract at 300 mg/kg. b.wt. [D] showing dilatation of portal vein (x80)

The representative picutures of histopathological examination of the liver tissue are shown in Fig. 5 (A-D) and the semi quantitative histological scoring of liver damage is presented in Table 5. Liver sections from the control group mice showed normal hepatic cytoarchitecture. They formed of hepatocytes radiating from central vein to the periphery of the lobules (Fig. 5A). Liver lobules of Cyp-treated mice showed severe degeneration in hepatocytes, necroses, dilation of portal vein with inflammatory cell infiltration in the portal area (Fig. 5B). Liver sections of mice treated with Cyp + Ext 150 (Fig. 5C) showed mild to moderate dilatation of central vein and ballooning degeneration in surrounding hepatocytes. Whereas, in cypermethrin + Ext 300 treated mice (Fig. 5C) showed only moderate dilatation of portal vein.

**Table 5:** The severity of the reaction in liver tissue of different groups according to the histopathological alterations.

Histopathological alterations	Treatment					
	Con	Ext 150	Ext 300	Cyp	Cyp+ Ext 150	Cyp+ Ext 300
Inflammatory cell infiltration in portal area	-	-	-	+++	-	-
Inflammatory cell infiltration in hepatic parenchyma	-	-	-	+++	-	-
Degeneration in hepatocytes	-	-	-	++++	++	-
Congestion in portal vein	-	-	-	++++	-	++
Congestion in central vein	-	-	-	+++	+	-

Con: control; Cyp: cypermethrin; Ext 150 & Ext 300; 150 and 300 mg/ kg b.wt of *C. grevei* MeOH extract. ++++ Very sever; +++ Sever; ++ moderate; + mild; - nil

## Discussion

The natural antioxidants present in many plants reduce oxidative damage and help prevent mutagenesis, carcinogenesis and aging due to their radical scavenging activities [8, 20]**.** Therefore, phenolic compounds of plants are very important because their hydroxyl groups confer antioxidant activity. Results of the present study showed that phenolics content of methanol and ethanol extract of C*. grevei* were similar quantities. However, no work in the literature cited the quantification of phenolic compound of that family. We have not detected flavonoids. These results are contradictory to those presented by the study of **Paris and Debray** [26]**,** which showed that the leaves of *C. grevei* contain 2 % flavonoids as glycosides of flavonols. The ethanol extract is the richest by tannins, followed by methanol extract. In addition, the ethanolic and methanol extracts have obtained intermediate values for anthocyanins [26]**.**

Free radicals are continuously produced in body and cause the oxidation of biomolecules (*e.g.*, protein, amino acids, lipid and DNA) which leads to cell injury and death. Our results revealed that EtOH and MeOH extract of *C. grevei* had DPPH scavenging activity in a concentration depending manner. *C. grevei* extracts (100 μg/mL) exhibited the highest inhibition of ABTS. In reducing power assay, Fe^3+^ was transformed to Fe^2+^ in the presence of MeOH and EtOH extracts of *C. grevei* to measure the reductive capability. *C. grevei* extracts had significant inhibition of reducing power with increasing concentration in the range of 0.10-0.125 mg/mL. The result obtained by this method confirms those of tests DPPH and ABTS.

In toxicological studies, changes in the body weight and relative organs weights are important criteria for evaluation of organ toxicity and were used as a valuable index of insecticide-related organ damage [8]. Liver is the first organ to face any foreign molecule that is carried through portal circulation and it is subjected to most damage. It has been previously reported that during liver damage there was an observed decrease in antioxidant defenses in the liver [26]. In the present study, the body weights of mice treated with Cyp was markedly less while relative liver weight was increased compared to the control group. Administration of *C. grevei* extract at 300 mg/kg b.wt. to Cyp-intoxicated mice restored body weight to normal range and modulated significantly relative live weight to the normal control value. The decrease in body weight of Cyp treated mice was considered the result of direct toxicity and/or indirect toxicity related to the liver damage [27–29]. It may be attributed to the effect of insecticides on gastrointestinal tract resulting in decreased appetite and absorption of nutrients from gut [30]. Other investigations have reported the reduction in body weight and change in relative organs weights in Cyp-treated rats [31, 32] and in rabbits [33]

Cyp treatment induced a severe hepatic damage in serum enzyme activities of ALT, AST, ALP and LDH of male mice. The increase in serum AST, ALT and ALP enzymes may be due to liver dysfunction and disturbance in the biosynthesis of these enzymes with alteration in the permeability of the liver membrane takes place [34]. The increase in serum LDH activity may be due to the hepatocellular necrosis leading to leakage of the enzyme into the blood stream [35]. However, co-administration of *C. grevei* extract to Cyp-treated mice resulted in a partial recovery in the above-mentioned parameters (AST, ALT, ALP and LDH). The high dose of *C. grevei* extract (300 mg/kg) was more effective than low dose (150 mg/kg) to attenuate the perturbations in the tested enzymes.

SOD and CAT are known to play an important role in scavenging ROS. SOD catalyzes the destruction of the superoxide radicals to H_2_O_2_, while CAT reduces the H_2_O_2_ into water and oxygen to prevent oxidative stress and in maintaining cell homeostasis. In addition, GST play essential role in the detoxification process. In the liver homogenates of Cyp-treated mice, the activities of SOD, CAT, GST and the level of GSH decreased significantly, while the level of LPO increased significantly. The change in SOD and CAT might be in response to increased oxidative stress. Considering that GST are detoxifying enzymes that catalyze the conjugation of a variety of electrophilic substrates to the thiol group of GSH, producing less toxic forms [36], the significant decrease of GST activity may indicate insufficient detoxification of Cyp in intoxicated mice.

Malondialdehyde (MDA) is a major oxidation product of peroxidized polyunsaturated fatty acids and increased MDA content is an important indicator of lipid peroxidation (LPO) [37]. In the present study, MDA level increased in mice exposed to Cyp. However, when a condition of oxidative stress strongly establishes, the defense capacities against ROS becomes insufficient, in turn ROS also affect the antioxidant defense mechanisms, reduces the intracellular concentration of GSH, alters the activity of antioxidant enzymes *e.g.*, SOD & CAT and increase MDA. These indirectly suggest an increased production of oxygen free radicals in mice. Highly reactive oxygen metabolites, especially hydroxyl radicals, act on unsaturated fatty acids of phospholipid components of membranes to produce malondialdehyde, an LPO product [38]. However, co-administration of *C. grevei* extract to Cyp-treated mice resulted in a partial recovery in the above-mentioned parameters in a dose dependent manner.

The histopathological changes in liver showed that exposure of mice to Cyp resulted in degenerative changes in the liver, including degeneration, necrosis, and inflammatory cells in the portal area. The biomarkers of liver dysfunction corroborated with the histopathological lesions observed in the current study. These observations indicated marked changes in the overall histoarchitecture of liver in response to Cyp, which could be due to its toxic effects primarily by the generation of reactive oxygen species causing damage to the various membrane components of the cell. Co-administration of C*. grevei* extract showed marked improvement in its histological structure in comparison to Cyp-treated group alone and represented by nil to moderate degree in inflammatory cell infiltration in portal area, degeneration in hepatocytes and congestion in the portal vein and central vein.

The present study has demonstrated that *C. grevei* exert have a hepatoprotective effect against Cyp-induced oxidative damage and hepatotoxicity in mice. Normalized antioxidant enzymes and reduction in MDA level are likely to be the major mechanisms by which *C. grevei* prevented development of the liver damage. Supporting this hypothesis, we observed significant increases in SOD, CAT, and GST enzyme activity and decreases in the levels of MDA in liver tissue concomitant with insignificant changes in liver serum dysfunction biomarkers (AST, ALT, ALP and LDH) of Cyp-treated mice by the administration of *C grevei* especially at high dose (300 mg/kg/day). This might be due to the antioxidant activity and hydroxyl radical scavenging effect of *C. grevei*, which sported by DPPH, ABTS scavenging activity and reducing power measurements. However, the antioxidant activity of an antioxidant compound have been attributed to various mechanisms, among which are prevention of chain initiation, binding of transition metal ion catalysts, decomposition of peroxides, prevention of continued hydrogen abstraction, reductive capacity and radical scavenging [38].

## Conclusion

In view of the data of the present study, it can deduce that Cyp caused oxidative damage and liver dysfunction in male mice. *C. grevei* extract has protective effects on Cyp-induced lipid peroxidation, oxidative stress and liver damage. *C. grevei* was found to be an effective antioxidant in many *in vitro* assays such as DPPH, ABTS and reducing power. These results indicated that administration of *C. grevei* is useful, easy, and economical to protect humans against pesticides toxicity. The results presented here can be considered as the first information on the hepatoprotective and antioxidant properties of *C. grevei* extracts.

## References

[1] Elliot M, Janes NF (1978). Synthetic pyrethroids a new class of insecticides. Chem Soc Rev.

[2] Cox C (1996). Insecticide factsheet, Cypermethrin. Journal of Pesticide Reform.

[3] Crawford MJ, Croucher A, Huston DH (1981). Metabolism of cis and trans-cypermethrin in rats. Balance and tissue retention study. Journal of Agriculture and Food Chemistry.

[4] Marei AEM, Ruzo LO, Casida JE (1982). Analysis and persistance of permethrin, cypermethrin, deltamethrin and fenvalerate in the fat and brain of treated rats. Journal of Agriculture and Food Chemistry.

[5] Desi I, Dobronyi I, Varga L (1986). Immuno-, neuro-, and general toxicologic animal studies on a synthetic pyrethroid: cypermethrin. Ecotoxicol Environ Saf.

[6] Baselt R (2008). Disposition of toxic drugs and chemicals in Man, 8th edition.

[7] Mansour SA, Mossa AH (2009). Lipid peroxidation and oxidative stress in rat erythrocytes induced by chlorpyrifos and the protective effect of zinc. Pestic Biochem Physiol.

[8] Mossa AT, Refaie AA, Ramadan A, Bouajila J (2013). Amelioration of prallethrin-induced oxidative stress and hepatotoxicity in rat by the administration of *Origanum majorana* essential oil. Biomed Research International.

[9] Mossa AH, Swelam ES, Mohafrash SMM. Sub-chronic exposure to fipronil induced oxidative stress, biochemical and histotopathological changes in the liver and kidney of male albino rats. *Toxicology Reports*. 2015; http://dx.doi.org/10.1016/j.toxrep.2015.02.009 10.1016/j.toxrep.2015.02.009 PMC5598362 28962413

[10] Gutteridge JM, Halliwell B (2010). Antioxidants: molecules, medicines, and myths. Biochem Biophys Res Commun.

[11] Mimić-Oka J, Simić T, Djukanović L, Reljić Z, Davicević Z (1999). Alteration in plasma antioxidant capacity in various degrees of chronic renal failure. Clin Nephrol.

[12] Nice D, Yalpani M (1997). Antioxidant based nutraceuticals. New technologies for healthy foods and nutraceuticals.

[13] Mulholland DA, Kotsos M, Mahomed HA, Randrianarivelojosia M (1999). The chemistry of the Ptaeroxylaceae. Nigerian Journal of Natural Products and Medicine.

[14] Afoulous S, Ferhout H, Raoelison GE, Valentin A, Moukarzel B, Couderc F (2013). Chemical composition and anticancer, antiinflammatory, antioxidant and antimalarial activities of leaves essential oil of *Cedrelopsis grevei*. Food Chem Toxicol.

[15] Rakotoarison O, Rabenau I, Lobstein A, Um BH, Schott C, Anton R (2003). Vasorelaxing properties and Bio-guided fractionation of *Cedrelopsis grevei*. Planta Med.

[16] Mulholland DA, Kotsos M, Mahomed HA, Koorbanally NA, Randrianarivelojosia M, van Ufford LQ (2002). Coumarins from *Cedrelopsis grevei* (Ptaeroxylaceae). Phytochemistry.

[17] Atmaca M, Bilgin HM, Obay BD, Diken H, Kelle M, Kale E (2011). The hepatoprotective effect of coumarin and coumarin derivates on carbon tetrachloride-induced hepatic injury by antioxidative activities in rats. J Physiol Biochem.

[18] Mossa AH, Heikal TM, Mohafrash SMM, Refaie AA (2015). Antioxidant potential and hepatoprotective activity of *Origanum majorana* leaves extract against oxidative damage and hepatotoxicity induced by pirimiphos-methyl in male mice. J Appl Sci.

[19] Conwell DG, Jones KH, Jiang Z, Lantry LE, Keely PSW, Kohar I (1998). Cytotoxicity of tocopherols and their quinines in drug-sensitive and multidrug-resistant leukemia cells. Lipids.

[20] Cook NC, Samman S (1996). Flavonoids chemistry, metabolism, cardioprotective effects, and dietary sources. J Nutr Biochem.

[21] Bruck R, Hershkoviz R, Lider O, Aeed H, Zaidel L, Matas Z (1996). Inhibition of experimentally-induced liver cirrhosis in rats by a non peptidic mimetic of the extracellular matrix associated Arg-Gly-Asp epitope. J Hepatol.

[22] El Kar C, Ferchichi A, Attia F, Bouajila J (2011). Pomegranate (*Punica granatum*) juices: chemical composition, micronutrient cations, and antioxidant capacity. J Food Sci.

[23] Oyaizu M (1986). Studies on product of browning reaction prepared from glucose amine. Jpn J Nutr.

[24] Tomlin CDS (2004). The e-Pesticide Manual, 13^th^ed.

[25] Michael JD (2008). The toxicologist’s pocket handbook. 2nd ed.

[26] Paris RR, Debray M (1972). Sur les polyphénols (acides-phénols, Flavonoïdes) des feuilles de deux Méliacées malgaches : *Cedrelopsis grevei* Baillon et Neobeguea Leroy. Plantes Méd Phyto.

[27] Seven A, Güzel S, Seymen O, Civelek S, Bolayirh M, Uncu M (2004). Effects of vitamin E supplementation on oxidative stress in streptozotocin induced diabetic rats: investigation of liver and plasma. Yonsei Med J.

[28] Abbassy MA, Mossa AH (2012). Haemato-biochemical effects of formulated and technical cypermethrin and deltamethrin insecticides in male rats. J Pharmacol Toxicol.

[29] Sankar P, Telang AG, Manimaran A (2012). Protective effect of curcumin on cypermethrin-induced oxidative stress in Wistar rats. Experimental Toxicology Pathology.

[30] Venkateshwarlu P, Sharma BJR, Kalakumar B, Reddy KS, Ravikumar P (1997). Comparative evaluation of toxicity of carbaryl, cypermethrin and malathion of testis in mice. Indian Journal of Toxicology.

[31] Hussain S, Khan MZ, Khan A, Javed I, Asi MR (2009). Toxico-pathological effects in rats induced by concurrent exposure to aflatoxin and cypermethrin. Toxicon.

[32] Sangha GK, Kaur K, Khera KS, Singh B (2011). Toxicological effects of cypermethrin on female albino rats. Toxicol Int.

[33] Lakkawar AW, Chattopadhyay SK, Somuanshi R (2004). Experimental cypermethrin toxicity in rabbits- a clinical and patho-anatomical study. Folia Veterinaria.

[34] De Bruin A (1979). Biochemical toxicology of environmental agents.

[35] Wang X, Zhai W (1988). Cellular and biochemical in bronchoalveolar lavage fluids of rats exposed to fenvalerate. Zhongguo Yaolixue Yu Dulixue Zoghi.

[36] Hayes D, Pulford D (1995). The glutathione S-transferase supergene family: regulation of GST and the contribution of the isoenzymes to cancer chemoprotection and drug resistance. Crit Rev Biochem Mol Biol.

[37] Kelly SA, Harvilla KM, Brady TC, Abrano KH, Leveir ED (1998). Oxidative stress in toxicology: established mammalian and emerging piscine model systems. Environmental Health Perspective.

[38] Mossa AH, Nawwar GA (2011). Free radical scavenging and antiacetylcholinesterase activities of *Origanum majorana* L. essential oil. Human & Experimental Toxicology.

